# Die Bedeutung des humanen Mikrobioms für die psychische Gesundheit

**DOI:** 10.1007/s00115-023-01552-x

**Published:** 2023-10-17

**Authors:** Alexander Refisch, Martin Walter

**Affiliations:** 1 Klinik für Psychiatrie und Psychotherapie, Universitätsklinikum Jena, Philosophenweg 3, 07743 Jena, Deutschland; 2 Deutsches Zentrum für psychische Gesundheit (DZP), Jena, Deutschland; 3 Center for Intervention and Research on adaptive and maladaptive brain Circuits underlying mental health (C-I-R-C), Jena, Deutschland

**Keywords:** Darm-Hirn-Achse, Immunometabolische Veränderungen, Neurotransmitter, Energiehomöostase, Neuronale Funktionen, Gut-brain axis, Immunometabolic dysregulation, Neurotransmitter, Energy homeostasis, Neural functions

## Abstract

Die Zusammensetzung des Mikrobioms ist bei vielen häufigen psychiatrischen Erkrankungen verändert. Präklinische Studien haben wichtige Mechanismen aufgedeckt, über die das Mikrobiom mit neuronalen Funktionen im bidirektionalen Austausch steht. Dysregulationen im komplexen Zusammenspiel von Mikrobiom, Immunsystem, Stress-Response und Energiehomöostase könnten insbesondere in der frühen Lebensphase für die Entwicklung psychiatrischer Symptome im späteren Leben prädisponieren. Obwohl bisher nur wenige klinische Studien vorliegen, haben der weitreichende Einfluss des Mikrobioms auf neuronale und psychische Funktionen sowie seine hohe Plastizität großes Interesse an seinem therapeutischen Potenzial bei häufigen psychiatrischen Störungen geweckt.

## Abstract (en)

Many common diseases including psychiatric disorders show characteristic alterations in the microbiome. Preclinical studies have uncovered important mechanisms by which the microbiome interacts bidirectionally with neural functions. Dysregulation of the complex interplay between the microbiome, immune system, stress response, and energy homeostasis, particularly in the early stages of life, can predispose to the development of psychiatric symptoms later in life. Although few clinical studies are available to date, the broad influence of the microbiome on neural and mental functions as well as its high plasticity, have generated great interest in its therapeutic potential for common psychiatric disorders.

## Hintergrund

Mikroorganismen besiedeln praktisch jeden Bereich unseres Planeten. Ihre Wechselwirkungen untereinander, mit anderen Lebewesen und ihrer Umwelt sind von grundlegender Bedeutung für die Funktion aller Ökosysteme, ein stabiles Klima sowie das Wohlbefinden von Pflanzen, Tieren und Menschen. Eine ungesunde Lebensführung oder infektiöse Mikroorganismen können dramatische Dysbalancen verursachen, die eine Vielzahl häufiger Krankheiten zur Folge haben.

In jüngster Zeit haben Forschungen gezeigt, dass humane Mikroorganismen, gemeinhin in ihrer Gesamtheit innerhalb eines Wirts als Mikrobiota oder bezogen auf die genetische Information als Mikrobiom bezeichnet, über direkte und indirekte Verbindungen in komplexer Wechselbeziehung zum zentralen Nervensystem (ZNS) stehen und somit Prädisposition, Entstehung und Aufrechterhaltung psychischer Symptome maßgeblich beeinflussen. Neue Erkenntnisse über diese bidirektionale Beziehung zwischen Mikrobiom und ZNS könnten das Verständnis heterogener, bisher unzureichend verstandener psychiatrischer Krankheitsbilder erweitern und den Weg für neue Biomarker und individualisierte Therapieansätze ebnen.

### Infobox

- Etwa 10^13^ Mikroorganismen besiedeln den menschlichen Körper.
- Das Verhältnis zwischen Mikroorganismen und Zellen beträgt in etwa 1,3:1.
- Allein im Darm vermutet man ca. 3,3 Mio. mikrobielle Gene im Vergleich zu 22.000 Genen im humanen Genom – die mikrobielle DNA ist zudem mit ca. einer Mutation alle 20 min hochvariabel und zeigt große interindividuelle Unterschiede.
- Die Zusammensetzung der Mikrobiota ist zudem extrem plastisch. Neben tageszeitlichen Schwankungen können nahezu sämtliche alltägliche Umwelteinflüsse wie Ernährung, Stress und körperliche Aktivität innerhalb von Stunden zu signifikanten Veränderungen führen.
- Enorme Fortschritte in der Sequenzierungstechnik konnten zeigen, dass selbst zuvor steril geglaubte Körperkompartimente, wie Lunge und Gehirn, ein charakteristisches ansässiges Mikrobiom aufweisen.
- Viele häufige Erkrankungen einschließlich psychischer Störungen sind mit charakteristischen Veränderungen im Darmmikrobiom assoziiert.
- Darüber hinaus steht das Mikrobiom in engem Zusammenhang mit verschiedenen physiologischen Eigenschaften, die für psychische Funktionen relevant sind, wie z. B. Neurotransmission, neuronale Plastizität, Stressregulation, Immunsystem und metabolische Faktoren.

## Mikrobiota-Hirn-Achse: Kommunikationswege im Zusammenhang mit psychischen Funktionen

**Figure 1:**
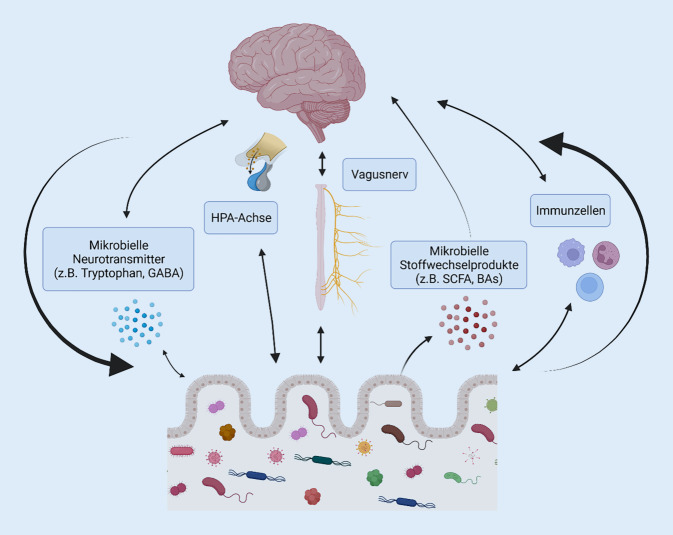

Die Redewendungen „Bauchgefühl“, „aus dem Bauch heraus“ und „Wut im Bauch“ existieren nicht nur im übertragenen Sinne. Der weit verbreitete Einsatz keimfreier (gnotobiotischer) Tiermodelle (GF) sowie von Tieren, deren Mikrobiom durch Antibiotikagabe, pathogene bakterielle Infektionen und fäkale Mikrobiotatransplantation (FMT) verändert wurde, hat es Mikrobiomforschern in den letzten 20 Jahren ermöglicht, die komplexen Signalwege und Mechanismen aufzudecken, über die menschliche Mikroorganismen in ständigem Austausch mit dem Gehirn stehen und u. a. Einfluss auf die psychische Gesundheit nehmen (Abb. 1).

### Das Mikrobiom und das Immunsystem

Die bidirektionale Verbindung mit dem Immunsystem ist einer der zentralen Mechanismen, über die Mikrobiota mit dem ZNS kommunizieren. Einerseits nehmen neuronale Schaltkreise Einfluss auf die Zytokinfreisetzung, um die Homöostase aufrechtzuerhalten und damit überschießende schädliche Entzündungsreaktionen zu verhindern [1], die sich sekundär auch auf die Zusammensetzung des Mikrobioms auswirken könnten [2]. Andererseits leistet das Mikrobiom über verschiedene, bisher noch weitestgehend unzureichend verstandene Mechanismen einen entscheidenden Beitrag in der Entwicklung und Reifung des Immunsystems [3]. So konnte in GF gezeigt werden, dass sich bestimmte Komponenten des Immunsystems nicht vollständig entwickeln können, was darauf hindeutet, dass einige wirtsspezifische Bakterien für die vollständige Reifung des Immunsystems unerlässlich sind [4]. Außerdem beeinflussen humane Mikroorganismen relative Populationen, Migration und Funktion diverser Immunzellen, einschließlich T‑Helfer-Zellen und regulatorischer T‑Zellen [5], die bei psychiatrischen Erkrankungen wie der unipolaren Depression mit einer veränderten Differenzierung assoziiert sind [6].

Insbesondere die ersten Lebensjahre, in denen die Immunzellen lernen, symbiontische Mikroorganismen zu tolerieren, scheinen für die Entwicklung und Schulung des Immunsystems entscheidend zu sein [7]. Interessanterweise finden fundamentale Ereignisse des Aufbaus der Wirtsimmunität parallel zu kritischen Zeitpunkten der neuronalen Entwicklung statt [8], in denen auch die Zusammensetzung des Mikrobioms die höchste intra- und interindividuelle Variabilität aufweist, bevor es etwa ab dem dritten Lebensjahr eine stabilere, erwachsenenähnliche Konfiguration erreicht [3]. Eine gestörte gegenseitige Wechselwirkung in dieser frühen Lebensphase, z. B. durch frühkindlichen Stress, könnte mit lang anhaltenden Auswirkungen auf verschiedene Aspekte der Immunhomöostase, einer erhöhten Anfälligkeit für Infektionskrankheiten und einer veränderten inflammatorischen Response verbunden sein, wie sie bei verschiedenen psychiatrischen Erkrankungen beobachtet wird [9].

Das Mikrobiom ist direkt in die Reifung und Funktion ZNS-spezifischer Immunzellen involviert

Abgesehen von der Etablierung von Komponenten des peripheren Immunsystems ist das Mikrobiom auch direkt in die Reifung und Funktion ZNS-spezifischer immunologischer Zellen involviert. So konnte im Vergleich zu konventionell aufgewachsen Tiermodellen gezeigt werden, dass GF eine erhöhte Anzahl unausgereifter Mikroglia mit veränderter Morphologie aufweisen, die mit einer beeinträchtigten Immunantwort einhergehen [10]. Die postnatale Supplementierung mit kurzkettigen Fettsäuren („short chain fatty acids“, SCFA) normalisierte erstaunlicherweise Form und Funktion der Mikroglia [11].

Darüber hinaus ist selbst ein vollständig ausgebildetes, gut trainiertes adultes Immunsystem weiterhin auf kommensale Mikroorganismen als erste Verteidigungslinie gegen pathogene Keime angewiesen. Ohne eine ausgewogene Balance in der Mikrobiomzusammensetzung als erste Verteidigungslinie, wäre der Wirt im ständigen Kampf gegen bakterielle Infektionen hoffnungslos unterlegen mit weitreichenden lokalen und systemischen Folgen [3]. Zum Beispiel werden chronische milde Entzündungen, die durch erhöhte Konzentrationen zirkulierender proinflammatorischer Zytokine und Akute-Phase-Proteine gekennzeichnet sind, mit zahlreichen häufigen Erkrankungen einschließlich psychischer Störungen in Verbindung gebracht, wobei die Ursachen noch weitgehend unklar sind [12]. Eine mögliche Quelle ist die sog. „Leaky-gut-Theorie“, nach der bakterielle Antigene durch eine erhöhte Permeabilität des Darmepithels leichter in die Blutbahn gelangen und eine systemische Entzündungsreaktion auslösen [20]. Das Darmmikrobiom spielt bei der Aufrechterhaltung dieser selektiven Permeabilität eine grundlegende Rolle, indem mikrobielle Metaboliten wie SCFA und sekundäre Gallensäuren („bile acids“, BAs) u. a. die Homöostase des Darmepithels gewährleisten und die Schleimsekretion im Kolon regulieren [13].

Interessanterweise konnte die lange vorherrschende Meinung eines immunprivilegierten autonomen ZNS, das durch die Blut-Hirn-Schranke (BHS) vom peripheren Immunsystem isoliert ist, kürzlich revidiert werden [14]. Mit der Entdeckung lymphatischer Gefäße innerhalb der Dura mater [15] und durch periphere Zytokine ausgelöste Signalkaskaden, die ein Aufbrechen der Tight Junctions in den Epithelzellen der BHS mit daraus resultierender erhöhter Permeabilität bewirken können [16], haben Forscher erst begonnen den Einfluss peripherer Mechanismen auf neuroinflammatorische Prozesse zu entschlüsseln [17], die zunehmend als Schlüsselfaktor für das Auftreten psychischer Symptome angesehen werden [18]. Ähnlich wie auf der Ebene des Darms nimmt das Mikrobiom auch in hierarchisch übergeordneten Bereichen maßgeblich Einfluss auf die immunoinflammatorischen Vorgänge. Zum einen ist inzwischen erwiesen, dass SCFA in die Wiederherstellung von Proteinen des Tight-Junction-Komplexes involviert und so für die Erhaltung der Integrität der BHS unter pathologischen Bedingungen essenziell sind [19]. Zum anderen exprimieren periphere Lymphozyten vielfach spezifische Rezeptoren für Stoffwechselprodukte humaner Mikroorganismen [20], die die Rekrutierung aus dem systemischen Kreislauf ins ZNS regulieren [21], was den Zusammenhang zwischen Mikrobiom, Immunsystem und Mechanismen, die psychiatrischen Symptomen zugrunde liegen könnten, weiter untermauert.

Zwischen parodontalen und psychischen Erkrankungen besteht eine hohe Komorbidität

Neben dem Darmmikrobiom ist in diesem Zusammenhang auch das orale Mikrobiom von besonderem Interesse. Zum einen besteht eine hohe Komorbidität zwischen parodontalen und psychischen Erkrankungen [22]. Durch die lokale Entzündungsreaktion gelangen ortsspezifische, symbiontische oder pathogene Mikroorganismen leichter in die Blutbahn [23], was es ihnen ermöglicht, sich in anderen „fremden“ Nischen des Körpers anzusiedeln und dort schädliche Wirkungen hervorzurufen. Ein eindrucksvolles Beispiel ist die infektiöse Endokarditis, die durch *Streptococcus viridans *verursacht wird, der als Bestandteil des kommensalen oralen Mikrobioms nach einer hämorrhagischen oralen Behandlung wie einer Zahnextraktion über die Blutbahn zum Herzen gelangt [24]. Zum anderen ist das orale Mikrobiom aufgrund der räumlichen Nähe zum Gehirn in Bezug auf immunoinflammatorische Interaktionen mit dem ZNS von besonderem Interesse. So gelangt das Blut aus der Mundhöhle im Gegensatz zu den Gefäßen des Gastrointestinaltraktes nicht zuerst in die Leber, wo mikrobielle Stoffwechselprodukte über einen „First-pass-Effekt“ bereits metabolisiert werden, sondern direkt zur BHS mit einem möglichen Einfluss auf relevante neuronale Funktionen, die zur Manifestation psychiatrischer Symptome beitragen könnten.

### Das Mikrobiom und die Verarbeitung von Stress

Die Hypothalamus-Hypophysen-Nebennierenrinden(HPA)-Achse ist vor allem für ihre Rolle bei der Vermittlung der Stressreaktion bekannt. Eine Störung der HPA-Achse ist einer der meist replizierten pathophysiologischen Befunde bei diversen psychiatrischen Erkrankungen, insbesondere der unipolaren Depression [25]. Chronischer Stress ist mit einer reduzierten Diversität im Mikrobiom assoziiert [26]. Umgekehrt hat das Darmmikrobiom einen großen Einfluss auf die Entwicklung der HPA-Achse, wie eine bahnbrechende Studie aus dem Jahr 2004 zeigt [27]. Sudo und Mitarbeiter konnten nachweisen, dass GF im Vergleich zur Kontrollgruppe auf milden Stress mit einer erhöhten Ausschüttung von adrenokortikotropem Hormon (ACTH) und Kortikosteron reagieren. Die verstärkte Stress-Response konnte in den GF teilweise durch eine FMT der Kontrollgruppe als Donor und vollständig durch die Supplementation eines einzelnen bakteriellen Stamms (*Bifidobacterium infantis*) wieder revidiert werden [27]. Interessanterweise war die Umkehr der Stress-Response in den GF-Modellen durch FMT oder Gabe des Probiotikums zeitabhängig, was erneut auf einen kritischen Zeitpunkt für die Wechselwirkung zwischen Mikrobiom und Wirt in der Entwicklung elementar physiologischer Funktionen, die die Vulnerabilität für psychische Erkrankungen beeinflussen, hindeutet.

Der Vagusnerv ist ein zentraler Kommunikationsweg der zwischen Darmmikrobiom und Gehirn

Ein möglicher Mechanismus besteht neben der mikrobiellen Modulation der zentralen Genexpression von Rezeptoren relevanter Neurotransmitter im Zusammenspiel mit weiteren wesentlichen Risikofaktoren, die für psychiatrische Symptome prädisponieren, wie frühkindlicher Stress, metabolische Störungen und einer veränderten inflammatorischen Response. Dysregulationen in diesen komplexen Wechselwirkungen könnten die Manifestation psychiatrischer Symptome begünstigen oder bei vulnerablen Patientinnen und Patienten sogar verursachen.

Nicht zuletzt trägt Stress zu einer Verschiebung des sympathovagalen Gleichgewichts zulasten der parasympathischen Modulation bei, was für viele psychiatrische Erkrankungen konsistent beschrieben wurde [28]. Der Vagusnerv, dessen Stimulation u. a. auch antiinflammatorische Effekte vermitteln kann, ist ein zentraler Signalweg der bidirektionalen Kommunikation zwischen Darmmikrobiom und Gehirn. Periphere vagale afferente Fasern respondieren einerseits auf Veränderungen in der Mikrobiomkomposition sowie direkt auf bakterielle neurometabolische Stoffwechselprodukte und leiten diese weiter zum ZNS [29]. Wichtige Erkenntnisse über die Rolle des Vagusnervs bei der Signalübermittlung zwischen Mikrobiom und Gehirn konnten durch Studien gewonnen werden, bei denen eine Vagotomie bei Tiermodellen erfolgt ist. Zum Beispiel konnten Bravo und Kollegen nachweisen, dass zuvor nachgewiesene positive Effekte des Probiotikums *Lactobacillus rhamnosus *auf ängstliches Verhalten und die Expression zentraler GABA-Rezeptoren in vagotomierten Mäusen nicht zu beobachten waren [30].

### Das Mikrobiom und die Energiehomöostase

Viele psychiatrische Erkrankungen gehen mit einem subjektiven Gefühl der Kraft- und Energielosigkeit einher, was sich auch objektiv in einer geringeren körperlichen und kognitiven Leistungsfähigkeit abbildet. Zudem besteht ein bidirektionaler Zusammenhang zwischen Adipositas und psychischer Gesundheit, welcher nur unzureichend durch Psychopharmakotherapie und einen ungesunden Lebensstil erklärt werden kann [31]. Umgekehrt konnte gezeigt werden, dass eine FMT von Patientinnen mit Anorexia nervosa zu einer verminderten Gewichtszunahme bei GF-Mäusen führt [32]. Diese Befunde deuten auf eine veränderte Energiehomöostase als wichtigen zugrunde liegenden biologischen Faktor hin, der entscheidend zur Entstehung und Aufrechterhaltung psychiatrischer Erkrankungen beitragen kann.

Durch die Verdauung komplexer Makronährstoffe, für die der menschliche Darm nicht die volle enzymatische Kapazität besitzt, sind humane Mikroorganismen direkt in die Regulation der Energieaufnahme involviert. Daneben nehmen Mikrobiota indirekt auf verschiedenen Ebenen der Mikrobiom-Hirn-Achse Einfluss auf die Vermittlung von Hunger- und Sättigungssignalen [33].

Es besteht ein bidirektionaler Zusammenhang zwischen Mikrobiom und Mitochondrien

Darüber hinaus besteht ein bidirektionaler Zusammenhang zwischen dem Mikrobiom und Mitochondrien, die beide von den primitiven α‑Proteobakterien abstammen und mütterlicherseits vererbt werden. Das Mikrobiom ist für die Produktion eines großen Anteils bioaktiver Moleküle verantwortlich, die für wichtige Stoffwechselfunktionen in den Mitochondrien wie Glykolyse, Zitratzyklus oder oxidative Phosphorylierung essenziell sind [34]. Da das Gehirn eines durchschnittlichen Erwachsenen im Ruhezustand 20 % der Energie des Körpers verbraucht [35], um den hohen Energiebedarf für die synaptische Signalübertragung und bei der Neuroproliferation zu decken [36], ist es sehr anfällig für Bedingungen, die mit einer gestörten Energieproduktion zusammenhängen. Entsprechend geht eine mitochondriale Dysfunktion mit einer verminderten zellulären Resilienz und Neuroplastizität einher, die für psychische Erkrankungen prädisponieren könnte [37].

Interessanterweise können humane Mikroorganismen auch das Verlangen des Wirts auf bestimmte Lebensmittel, wie z. B. Kohlenhydrate, beeinflussen, um sich dem Selektionsdruck zu widersetzen und ihr eigenes Überleben zu sichern. Heißhunger auf Kohlenhydrate wurde immer wieder mit Veränderungen der Stimmung in Verbindung gebracht, im Sinne einer Art Selbstmedikation zur Aufhellung eines Stimmungstiefs [38–40]. Darüber hinaus konnte gezeigt werden, dass eine positive Korrelation zwischen stärkerem Verlangen nach Kohlenhydraten und der Stimmung besteht [41]. Umgekehrt wirkt sich eine dysregulierte Kohlenhydratzufuhr negativ auf den Antrieb aus [42]. Wenn Patientinnen oder Patienten im Rahmen emotionaler Dysbalance auf kohlenhydratreiche Nahrungsmittel zurückgreifen, verstärken sie somit mikrobielle, entzündliche, metabolische und hormonelle Veränderungen, die psychiatrischen Symptomen zugrunde liegen können. Daher könnte das Verlangen nach Kohlenhydraten bei psychischen Erkrankungen sowohl ein Kompensationsmechanismus als auch ein bedingender und aufrechterhaltender Faktor der Störung zu sein, der ebenfalls durch das Mikrobiom beeinflusst wird.

### Das Mikrobiom und die Neurotransmitterproduktion

Es ist bemerkenswert, dass humane Mikroorganismen und Wirt eine gemeinsame neurochemische Sprache verwenden, mit der sie miteinander und untereinander kommunizieren. So ist seit langem bekannt, dass kommensale Mikroben in der Lage sind, Neurotransmitter und Neuromodulatoren direkt zu produzieren, die im gesamten menschlichen Körper breite Anwendung finden. Zum Beispiel produzieren *Lactobacillus spp.* und *Bifidobacterium spp.* Gamma-Aminobuttersäure (GABA), *Escherichia spp*., *Bacillus spp*. und *Saccharomyces spp.* Noradrenalin, *Candida spp., Streptococcus spp., Escherichia spp.* und *Enterococcus spp.* Serotonin, *Bacillus spp.* Dopamin und *Lactobacillus spp.* Acetylcholin [43]. Demzufolge stellen diese mikrobiellen neuroaktiven Verbindungen einen potenziellen Mechanismus dar, durch den unser Mikrobiom direkt mit den Nervenzellen des Wirts interagiert. Natürlich sind die von den Bakterien produzierten Mengen relativ gering, und es ist daher fraglich, ob sie einen signifikanten Einfluss auf die Neurotransmission beim Menschen haben. Dennoch könnten selbst kleine Mengen Prozesse beeinflussen, die für die psychische Gesundheit relevant sind. So konnte gezeigt werden, dass das Probiotikum *Lactobacillus rhamnosus*, das in Tiermodellen ängstliche und depressive Verhaltensweisen vermindert, auch die Expression des GABA-Rezeptors in verschiedenen Hirnregionen, einschließlich des Kortex, Hippokampus und Amygdala moduliert [30] und das Sozialverhalten positiv beeinflusst [44].

**Figure 2:**
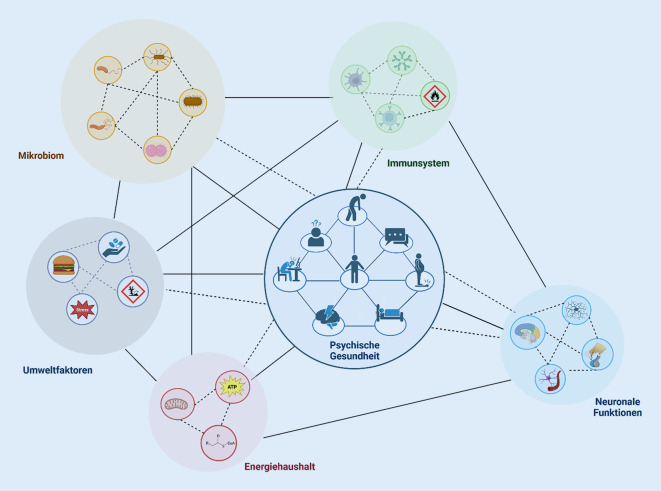

Serotonin, das vor allem aufgrund der viel beachteten und inzwischen revidierten Serotoninhypothese für affektive Erkrankungen [45] tendenziell als primär ZNS-bezogenes Molekül angesehen wird, wird tatsächlich zu mehr als 90 % im Darm synthetisiert. Serotonin selbst kann die BHS nicht passieren, im Gegensatz zu seinem Vorläufermolekül Tryptophan (TRP). TRP wird im Gehirn u. a. über das Enzyme Indolamin‑2,3‑Dioxygenase (IDO) zum N‑Methyl-D-Aspartat(NMDA)-Rezeptor-Antagonisten Kynurenin metabolisiert. Dysregulationen an dieser Schnittstelle zwischen Serotonin- und Glutamatstoffwechsel könnten zu weitreichenden Beeinträchtigungen neuronaler Funktionen führen und werden mit verschiedenen neuropsychiatrischen Erkrankungen wie Depression [46] und Alzheimer-Demenz [47] in Verbindung gebracht. Interessanterweise wird IDO als eine entscheidende Stellschraube im TRP-Stoffwechsel maßgeblich durch eine Entzündungsreaktion bzw. durch chronischen Stress (HPA-Hyperaktivität) induziert [48], die, wie in den vorangegangenen Kapiteln beschrieben, eng mit Veränderungen des Mikrobioms assoziiert sind. So konnte gezeigt werden, dass die TRP-Konzentrationen im Plasma von GF erhöht und das Verhältnis von Kynurenin zu TRP erniedrigt waren [49], was daraufhin deutet, dass die Mikrobiota die zentrale serotonerge Neurotransmission entscheidend verändert. Insbesondere beim Menschen ist ein Zusammenhang dieser Mechanismen mit Veränderungen im Mikrobiom, gerade in seiner Bedeutung für psychische Erkrankungen, jedoch noch nicht sehr gut untersucht (Abb. 2).

## Ausblick

Diese Arbeit konzentriert sich auf erste Erkenntnisse und hieraus resultierende Hypothesen zu biophysiologischen Prozessen, durch die das Mikrobiom die psychische Gesundheit beeinflusst, ohne eine offensichtliche Interaktion mit Verhaltens- oder psychosozialen Faktoren zu berücksichtigen. Gerade diese Wechselwirkung, die zentralnervöse Funktionen und ihre Interdependenz auch im Kontext sozialer Interaktion oder andere (z.B. olfaktorische) Wege, über die die Zusammensetzung des Mikrobioms die soziale Interaktion beeinflussen kann, sollten Gegenstand zukünftiger Untersuchungen sein. Vieles deutet auch auf eine mögliche Dysregulation der komplexen Interaktion zwischen humanen Mikroorganismen, dem Immunsystem, der Energiehomöostase und der Stressantwort hin, insbesondere zu kritischen Zeitpunkten in der Entwicklung, die für häufige psychiatrische Störungen und komorbide somatische Erkrankungen prädisponieren. Seine enorme Plastizität macht das Mikrobiom besonders interessant als Ziel für alternative therapeutische Interventionen, z. B. durch Lebensstiländerungen oder die Supplementierung mit Prä- und Probiotika, nicht nur im Erwachsenenalter, sondern bereits in der Adoleszenz. Die Evidenzlage für eine Wirksamkeit derartiger naheliegender Interventionen ist jedoch, trotz beeindruckender Einzelstudien [50], im Allgemeinen noch schlecht [51].

Die absolute Mehrheit der Erkenntnisse über mikrobielle Einflüsse auf psychische Funktionen stammt bislang aus präklinischen Untersuchungen an Tiermodellen. Translationale Ansätze stecken dagegen noch in den Kinderschuhen mit relativ kleinen Probandenzahlen, fehlender statistischer Aussagekraft und bislang kaum replizierten Befunden. Die Forschung hierzu entwickelt sich jedoch rasant mit einer Zunahme von Untersuchungen mit großen Fallzahlen, longitudinalen Designs, effizienteren Sequenzierungstechniken, der Miteinbeziehung zusätzlicher Biomarker und unter Berücksichtigung wichtiger Störvariablen wie z. B. der Ernährung.

Trotz zahlreicher, zum Teil widersprüchlicher Ergebnisse konnte kürzlich in einer systematischen Übersichtsarbeit und Metaanalyse ein übergeordnetes, transdiagnostisches Muster eines veränderten Mikrobioms mit vermindertem Vorkommen bestimmter entzündungshemmender SCFA-produzierender Gattungen und einer Anhäufung proinflammatorischer Bakterien bei unipolarer Depression, bipolarer affektiver Störung, Schizophrenie und Angststörungen identifiziert werden [52]. Interessanterweise konnte zusätzlich ein positiver Zusammenhang zwischen den charakteristischen Veränderungen im Mikrobiom und der Symptomschwere bei affektiven Erkrankungen und der Schizophrenie gezeigt werden [53]. Ständig wachsende Erkenntnisse auf dem Gebiet haben dazu geführt, dass sich der Fokus der Forschung von der einfachen Identifizierung von Assoziationen zwischen Mikrobiom und Psychopathologie hin zu Untersuchungen zur Kausalität und Translation in die klinische Versorgung verschoben hat [54]. Bislang sind die Ergebnisse zu Probiotika und zur FMT jedoch noch zu begrenzt, um daraus Schlussfolgerungen für den klinischen Alltag ziehen zu können [53].

Mit dem Startschuss des vom Bundesministerium für Bildung und Forschung (BMBF) geförderten Deutschen Zentrums für Psychische Gesundheit (DZP) haben Wissenschaftlerinnen und Wissenschaftler verschiedener Disziplinen neuerdings ideale Voraussetzungen, entsprechende Forschungsvorhaben in der notwendigen Komplexität sowie anhand großer, longitudinaler Untersuchungskohorten umzusetzen. Einen besonderen Fokus auf die Wechselwirkungen zwischen Mikrobiom und psychischer Gesundheit legt dabei z. B. der mitteldeutsche DZP-Standort Halle-Jena-Magdeburg (www.C-I-R-C.de) in enger Zusammenarbeit mit dem Exzellenzcluster „Balance of the Microverse“ der Universität Jena.

Neue Erkenntnisse über den Einfluss mikrobieller, immunologischer und metabolischer Veränderungen auf zentrale Schaltkreise versprechen daher nicht nur, häufige psychiatrische Störungen biologisch besser zu charakterisieren und individuellere Behandlungsstrategien zu entwickeln, sondern auch z. B. komorbiden Herz-Kreislauf- oder metabolischen Erkrankungen vorzubeugen.

## Fazit für die Praxis

- Psychiatrische Erkrankungen sind mit charakteristischen Veränderungen im Mikrobiom assoziiert.
- Das Mikrobiom beeinflusst physiologische Funktionen, die mit der Ätiopathogenese psychiatrischer Symptome in Verbindung gebracht werden.
- Dysregulationen in der Interaktion zwischen Mikrobiom, Immunsystem und neuronaler Entwicklung in der frühen Lebensphase prädisponieren für psychiatrische Erkrankungen.
- Fortschritte in der Sequenzierungstechnik ermöglichen effizientere Analysen des Mikrobioms.
- Seine enorme Plastizität macht das Mikrobiom zu einem vielversprechenden Target für therapeutische Interventionen zur Behandlung psychischer Erkrankungen.
